# Neuropeptide Y deficiency induces anxiety-like behaviours in zebrafish (*Danio rerio*)

**DOI:** 10.1038/s41598-020-62699-0

**Published:** 2020-04-03

**Authors:** Kazuhiro Shiozaki, Momoko Kawabe, Kiwako Karasuyama, Takayoshi Kurachi, Akito Hayashi, Koji Ataka, Haruki Iwai, Hinako Takeno, Oki Hayasaka, Tomonari Kotani, Masaharu Komatsu, Akio Inui

**Affiliations:** 1 Department of Food Life Science, Faculty of Fisheries, Kagoshima University, Kagoshima, Japan; 2 The United Graduate School of Agricultural Sciences, Kagoshima University, Kagoshima, Japan; 3 Department of Pharmacological Sciences of Herbal Medicine, Graduate School of Medical and Dental Sciences, Kagoshima University, Kagoshima, Japan; 4 Department of Oral Anatomy and Cell Biology, Graduate School of Medical and Dental Sciences, Kagoshima University, Kagoshima, Japan

**Keywords:** Animal behaviour, Peptide hormones, Animal disease models, Zoology

## Abstract

Neuropeptide Y (NPY) controls energy homeostasis including orexigenic actions in mammalians and non-mammalians. Recently, NPY has attracted attention as a mediator of emotional behaviour and psychosomatic diseases. However, its functions are not fully understood. We established *npy* gene-deficient (NPY-KO) zebrafish (*Danio rerio*) to assess the relationship between NPY and emotional behaviours. The NPY-KO zebrafish exhibited similar growth, but *pomc* and *avp* mRNA levels in the brain were higher as compared to wild-type fish. NPY-KO zebrafish exhibited several anxiety-like behaviours, such as a decrease in social interaction in mirror test and decreased locomotion in black-white test. The acute cold stress-treated NPY-KO zebrafish exhibited anxiety-like behaviours such as remaining stationary and swimming along the side of the tank in the mirror test. Moreover, expression levels of anxiety-associated genes (*orx* and *cck*) and catecholamine production (*gr*, *mr*, *th1* and *th2*) were significantly higher in NPY-KO zebrafish than in wild-type fish. We demonstrated that NPY-KO zebrafish have an anxiety phenotype and a stress-vulnerability like NPY-KO mice, whereby *orx* and/or catecholamine signalling may be involved in the mechanism actions.

## Introduction

Mammalian neuropeptide Y (NPY) consists of 36 amino acids, and is expressed both peripherally and in numerous brain regions, including the hypothalamus, amygdala, hippocampus, nucleus of the solitary tract, locus coeruleus, nucleus accumbens and cerebral cortex^1^. Mammalian NPY not only acts as a vasoconstrictor by regulating blood pressure around peripheral nerves, but also as a regulator of food intake and emotional behaviour^2,3^. Centrally NPY and its related neurons control feeding behaviour, energy balance, anxiety^4^, learning and memory^5^, fear^3^ and locomotor activity^6^. Four human NPY receptors (Y1, Y2, Y4 and Y5) have been cloned and characterized, and have different functions. For example, Y1 and Y2 increase blood pressure, Y1 and Y5 increase appetite, and Y2 and Y4 decrease appetite^1^.

NPY has been implicated in human diseases, particularly psychiatric disorders such as depression. Low NPY levels are associated with negative emotional processing and major depressive disorder^7^. NPY levels are low in the cerebrospinal fluid and platelet-poor plasma of depressed patients and suicide victims^8^. NPY polymorphisms, such as the −399G allele in the NPY promoter region, have been implicated in anxiety and depression^9^. Several rodent models have been used to study the mechanism underlying NPY-related psychiatric disorders, and the observed phenotypes are similar to those of human anxiety; as a result, NPY and its receptors have been targeted for depression therapy. Injecting NPY and/or NPY receptor antagonists into mouse brains has revealed the significance of NPY in depression. However, the functions of NPY are not fully understood because studies that have used different animal models have obtained dissimilar results, partly because of the methodology involved. Injecting NPY into the brain requires skill, and the dosage of injected exogenous NPY has sometimes been higher than the endogenous concentration. Recently, lines of NPY- and NPY-receptor-knockout (KO) mice have been established, and their phenotypes studied. NPY-KO mice suppress food intake after 48 h of fasting, decrease time spent in a central area and frequency of the rearing in open field test^6^. NPY Y1-KO mice exhibit decreased time spent in the lit compartment in light and dark test and decreased distance travelled in the central area of open field test^10^. NPY-KO animals can be used to study anxiety behaviour over a long time period with physiologically realistic NPY concentrations *in vivo*.

Recently, animal models other than rodents have been developed. For example, zebrafish (*Danio rerio*) are used in research on human diseases such as cancer, obesity and genetic disorders, as well as on social behaviour^11–14^. Zebrafish are relatively cheap to maintain, and need less space than rodents. In addition, whole genomic information is available for zebrafish^15^, and gene modification techniques, including genome editing, have been performed on the species. Zebrafish lay over 100 eggs a day, and their clear embryos and larvae can easily be observed. Drugs can easily be administered to zebrafish by simply supplementing the aquarium water. Similar analytical methods of studying anxiety to those for mice have been developed for zebrafish, such as open field tank, black-white preference and t-maze tests^16^. Therefore, the zebrafish is an excellent animal model for drug development^17^, first-medicine screening^18^, and studies on social behaviour^19^. The NPY amino acid sequence is strictly conserved in vertebrates such as birds, reptiles, amphibians and fish. Zebrafish NPY amino acid sequences have high similarity with human NPY (89%), and several studies have suggested that zebrafish NPY functions in a similar way as human NPY^20–22^.

However, NPY functions in zebrafish are not fully understood. Intracerebroventricular Administration (ICV) of exogenous NPY into the zebrafish brain results in an increase in food intake and a decrease in locomotor activity^23^. NPY agonists and antagonists would be useful to estimated fish NPY functions, and several mammalian NPY antagonists have been developed. In zebrafish, ICV of NPY increases time spent in the bright white environment, a normally aversive region, in black-white preference tests that is decreased by BIBP-3226, which is a mammalian Y1 antagonist^23^. In mammals, the Y1 receptor is anxiolytic, whereas the Y2 receptor is anxiogenic. The anxiolytic effects of Y4 receptor deletion are amplified by a Y2/Y4 receptor double-KO, suggesting a positive interaction between the Y2 and Y4 receptors. On the other hand, fish NPY receptors have been identified as Y1, Y2, Y2-2, Y4, Y7, Y8a and Y8b, but mammalian Y5 and Y6 are not conserved^24^. Therefore, NPY receptor antagonists that were designed for humans may provide little or misleading information in zebrafish.

We wondered whether NPY deficiency would affect emotional behaviours in zebrafish. In order to elucidate zebrafish NPY functions and assess the possibility of using zebrafish to study NPY-related diseases, we established NPY-knockout zebrafish (NPY-KO) using CRISPR/Cas9 genome editing. As zebrafish are oviparous, microinjection of a Cas9/gRNA complex into a one-cell-stage of fertilized egg is a straightforward procedure. After establishing NPY-KO zebrafish, their behaviour and stress-related gene expression were analysed.

## Results

### Establishment of NPY-knockout zebrafish

**Figure 1:**
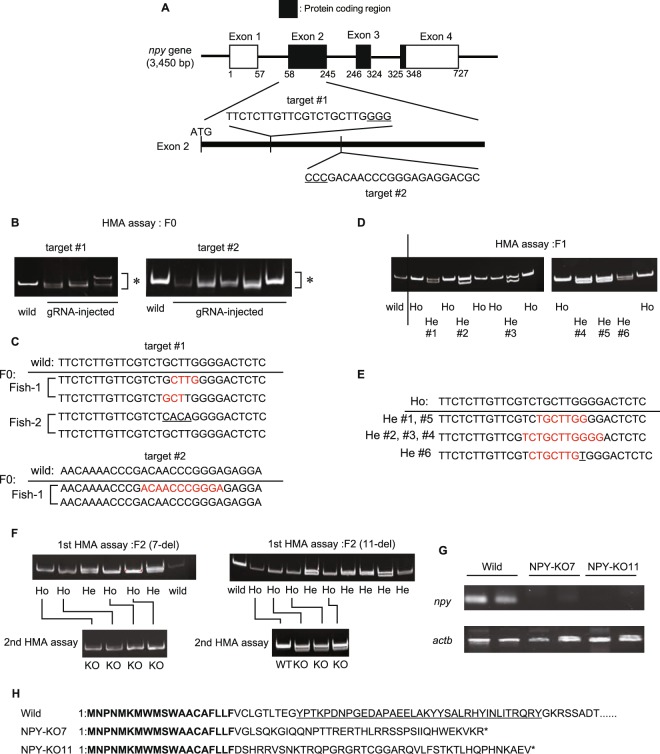
Establishment of NPY-knockout zebrafish. (**A**) The target sequence of guide RNAs (gRNAs) for zebrafish *npy*. Protein-coding exons are indicated as black boxes. Protospacer-adjacent motif (PAM) sequences are underlined. (**B**) F0 founder genotyping. An asterisk indicates the induction of heteroduplexes of the *npy* genome in gRNA-injected zebrafish. (**C**,**E**) *npy* nucleotide sequences around a gRNA target sequence in F0 (**C**) and F1 (**E**). Red coloured and underlined characters indicate a deletion and insertion in *npy* nucleotides, respectively, in comparison with wild-type zebrafish. Results of the nucleotide sequencing from two fish were shown as a representative in (**C**). Numbers in (**E**) are sample IDs that are used in (**D**). (**D**) F1 genotyping by heteroduplex mobility assay (HMA). Ho and He indicate homozygous and heterozygous alleles, respectively. (**F**) F2 genotyping. 1st HMA was conducted to distinguish homozygous and heterozygous. 2nd HMA was done to identify KO. (**G**) Expression of intact *npy* mRNA in wild-type fish and NPY-KO mutants. cDNAs from wild-type fish and mutants were used for PCR as a template, using primers specific to intact *npy*. *actb* was used as an internal control. (**H**) Deduced NPY amino acid sequences obtained from NPY-KO7 and NPY-KO11 nucleotide sequences. Bolding indicates conserved regions in wild-type fish and mutants. A mature NPY sequence is underlined, and asterisks indicate stop codons. Full-length gels are presented in Supplemental Fig. 2.

When establishing NPY-KO zebrafish, *npy*-specific guide RNAs (gRNAs) were designed for the zebrafish genome using CRISPRdirect^25^ to avoid off-targeting. Two gRNA candidates downstream of the first ATG were selected, target #1 was 5′-TTCTCTTGTTCGTCTGCTTGGGG-3′ and target #2 was 5′-CCCGACAACCCGGGAGAGGACGC-3′, and gRNAs were synthesized according to each target sequence (Fig. 1A and Supplemental Fig. 1). After microinjection of each gRNA/tracrRNA/rCas9 in one-cell-stage zebrafish embryos, genomic DNA was extracted from F0 embryos, and efficacy gRNAs for *npy* editing were estimated by conducting a heteroduplex mobility assay (HMA). Both gRNA#1- and gRNA#2-injected embryos showed double or triple bands, with a band shift with high efficacy (77.7 and 85.2% embryos with mutation in 27 gRNA-injected embryos, respectively), while a single band of a *npy* fragment was detected in wild-type embryos (Fig. 1B). These band patterns indicated the presence of various heterozygous *npy* alleles with mutation(s). DNA sequencing these HMA products revealed *npy* mutations in each RNA-injected embryo, confirming that *npy* genome editing by gRNAs was successful (Fig. 1C). As both gRNAs had high efficacy in inducing *npy* mutations, gRNA#1 was used for the establishment of the *npy*-knockout strain.

The F0 founder was crossed with wild-type zebrafish to obtain the F1 generation (Fig. 1D). Two F1 pairs had the same type of *npy* mutation (7- and 11-base deletion) on one allele, and were selected using DNA sequencing to establish the F2 generation (Fig. 1E). F2 generations was co-housed before genotyping. The establishment of F2 with homozygous *npy* alleles with mutation was confirmed by HMA assay (Fig. 1F) and DNA sequencing of *npy* cDNA from the F2 also confirmed the 7- or 11-base deletion (Supplemental Fig. 3). In addition, cDNA from brain RNA was subjected to polymerase chain reaction (PCR) analysis with primers specific to intact *npy*, but not to the *npy* mutant (Fig. 1G). The expected PCR bands were found in wild-type fish but not in 7- and 11-base-deletion mutants. The 7- and 11-base deletion in *npy* caused a frame shift, and resulted in the induction of polypeptides that were different to mature zebrafish NPY (Fig. 1H). As the 7- and 11-deletions were present in the same exon with the region encoding Npy mature peptide, unexpected splicing valiant in 7- and 11-deletion mutant could not yield Npy mature peptides (Supplemental Fig. 1).

**Figure 2:**
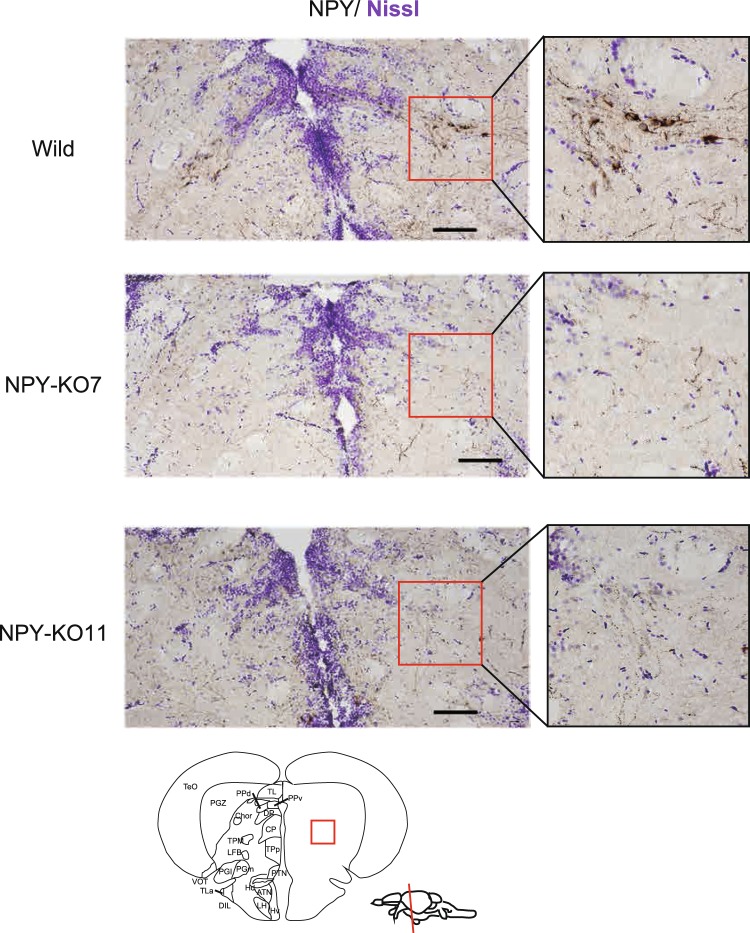
Expression of NPY in wild and NPY-KO zebrafish brain. Distribution of NPY-neurons in wild and NPY-KO zebrafish brain. NPY signals were immuno-stained by NPY-specific antibody and DAB staining. Neuronal cells were detected by Nissl staining. The area around the central posterior thalamic nucleus (CP) were highlighted with a black circle. Black bar indicates 100 μm.

By immunohistochemical analysis, NPY-positive neurons were detected around the central posterior thalamic nucleus (CP) in wild type zebrafish brain (Fig. 2), same as reported elsewhere^21^, while NPY-positive neurons were barely detected in wild type hypothalamus (Supplemental Fig. 4). Like no transcription of *npy* gene in NPY-KOs, NPY-positive neurons were not detected around the CP and in the hypothalamus of NPY-KO7 and KO11 (Fig. 2 and Supplemental Fig. 4). Weak signals detected in NPY-KO specimen would be due to artefact staining because there are not an alternative splicing isoform, other *npy* paralogs and other start codon inducing in-frame in NPY-KO mutants. As a result, *npy*-knockout zebrafish (NPY-KO) was established with a 7- or 11-base deletion and named NPY-KO7 and NPY-KO11, respectively.

**Figure 3:**
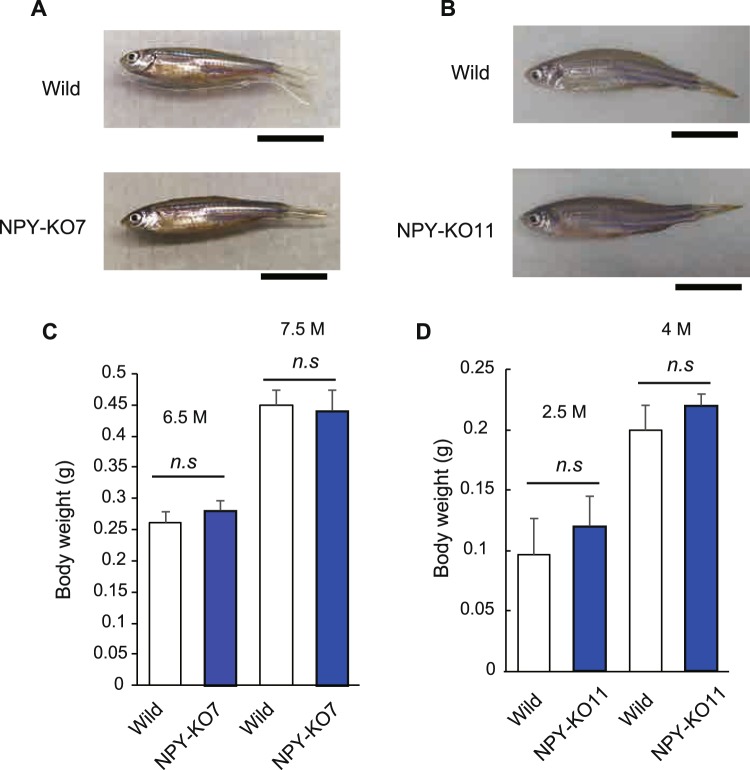
Growth in NPY-knockout zebrafish. (**A**,**B**) Morphological observations of wild-type and NPY-knockout zebrafish. Black bar indicates 1 cm. (**C**,**D**) Body weight of wild-type and NPY-knockout zebrafish; NPY-KO7 at 6.5 and 7.5 months old, and NPY-KO11 at 2.5 and 4 months old.

F2 genotyping by HMA revealed that the number of wild-type (+/+): hetero (+/−): knockout (−/−) fish was 23:44:18 and 24:16:13 in NPY-KO7 and NPY-KO11, respectively. In addition, no abnormal embryonic development was observed in either line. No abnormal morphologies were observed in NPY-KO zebrafish during growth (Fig. 3A,B), and body weight were also unaffected (Fig. 3C,D). KO lines were sustainable as homozygotes.

### Mirror test

**Figure 4:**
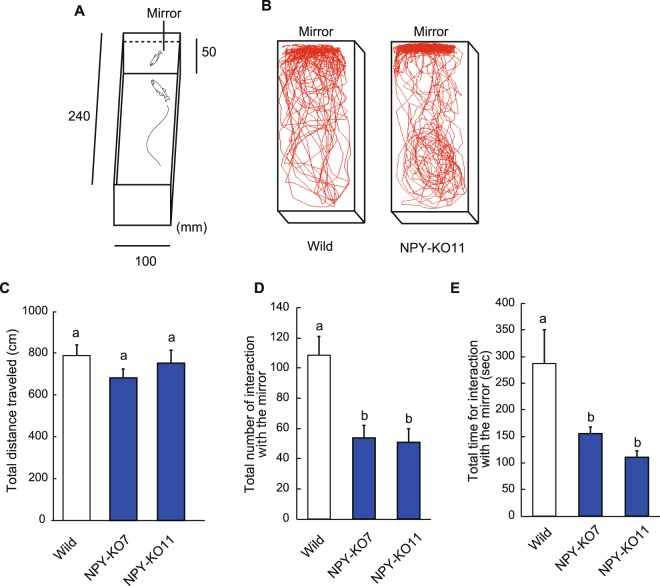
Analysis of NPY-KO and wild-type zebrafish behaviour. (**A**) Apparatus used in the mirror test. (**B**) Tracking of wild-type (left) and NPY-KO (right) zebrafish during analysis. (**C**) Total distance travelled. (**D**) Total number of interaction with the mirror. (**E**) Total time of interaction with the mirror. N = 15. Results are shown as means ± standard errors of three independent experiments. Columns having the same letter were not statistically different, *vice versa*.

The mirror test has been used as a method to assess the recognition of oneself in a mirror, sociality and aggression against their reflection in animals such as apes, elephant, dolphins, and social birds^26,27^. In zebrafish, the mirror test is an assay to measure aggressive behaviours^28^. The NPY-KO zebrafish underwent a mirror test to ascertain whether NPY deficiency affects behaviour, such as aggression and social interactions. In mirror tests, fish infers that another fish is present, approaches it and follows the opponent in the mirror (interaction with the mirror) (Fig. 4A). During the test period, total distance travelled did not differ among wild and NPY-KOs (Fig. 4B,C). On the other hand, the number of interaction with the mirror by NPY-KO7 and NPY-KO11 zebrafish was significantly lower than that by wild-type fish (*p* < 0.01), indicating that NPY-KO zebrafish were less sociable than wild-type fish (F = 35.63, *p* < 0.0001, one-way ANOVA; Fig. 4D). Approaching a mirror is also a pre-fight behaviour in zebrafish, and the total time interaction with the mirror also significantly decreased in NPY-KO compared with wild-type zebrafish (*p* < 0.01 in NPY-KO7 and KO11) (F = 18.01, *p* < 0.0005, one-way ANOVA; Fig. 4E). These results suggest that NPY deficiency causes a decrease in social interactions.

### Cold stress

**Figure 5:**
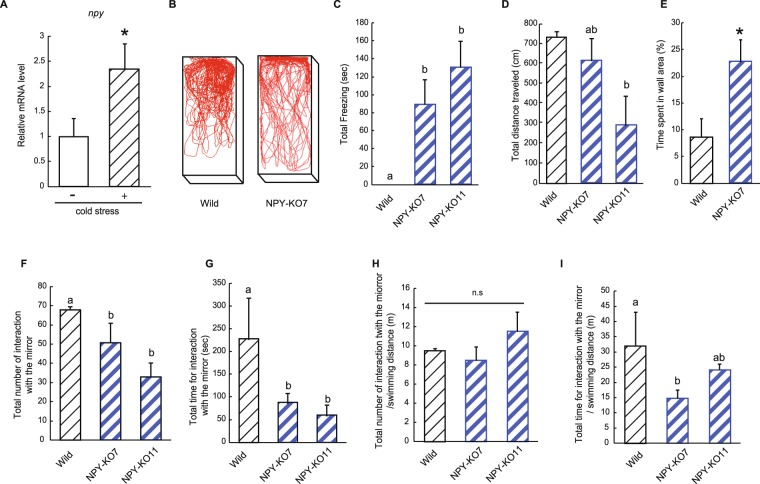
Changes in swimming behaviour in NPY-KO zebrafish under acute stress. (**A**) Changes in *npy* mRNA levels in wild-type zebrafish under cold stress. (**B**) Tracking of wild-type (left) and NPY-KO (right) zebrafish swimming behaviour after acute cold shock. (**C**) Total time spent stationary (frozen). (**D**) Total distance travelled. (**E**) Total time spent by the sides of the tank. (**F**) Total number of interaction with the mirror (**G**) Total time for interaction with the mirror. (**H**) Number of interaction to the mirror/swimming distance (m). (**I**) Time for the interaction/swimming distance (m). N = 15. Results are shown as means ± standard errors of three independent experiments. *p < 0.05, **p < 0.01. *n.s*., not significant. Columns having the same letter were not statistically different, *vice versa*.

In mice, physical and emotional stressors induce the elevation of plasma NPY and NPY mRNA transcription to reduce stress via corticoid receptor signalling^29^. In zebrafish and rainbow trout (*Oncorhynchus mykiss*), handling stress and high stocking densities increase *npy* mRNA upregulation^30,31^. To ascertain how *npy* deficiency affects zebrafish behaviour including social behaviour under stressful conditions, we conducted a cold-stress test, which is general stress in fish^32^. Surprisingly, long-term cold stress caused fatality in NPY-KO zebrafish but not wild-type fish in the preliminary experiments. Consequently, it was difficult to record zebrafish behaviour and gene expression levels under long-term cold conditions; however, we found a solution: fish were exposed to instantaneous cold stress (10 °C for 2 s) and then transferred to the mirror test tank. In wild-type zebrafish, the *npy* mRNA level was 2.4-fold higher under cold stress than in non-stressed zebrafish, confirming stress induction (*p* < 0.05, Fig. 5A). As shown in Fig. 5B, cold stress caused anxiety behaviour in the NPY-KO zebrafish. Freezing was observed in NPY-KO7 and NPY-KO11 zebrafish (80 and 120 s, respectively, *p* < 0.05), but not in wild-type fish (F = 18.43, *p* < 0.01, one-way ANOVA; Fig. 5C), inducing the significant reduction of swimming distance in NPY-KO (*p* < 0.05 in NPY-KO11) (F = 5.62, *p* < 0.01 one-way ANOVA; Fig. 5D). Regarding swimming behaviour, NPY-KO zebrafish exhibited abnormal swimming behaviour when moving along the side of the tank compared with wild type (Fig. 5B). Time spent by the tank side (excluding mirror side) was 2.4-fold higher in NPY-KO7 than wild-type zebrafish (*p* < 0.05, Fig. 5E). Time by the side was not estimated in NPY-KO11 because of long freezing (Fig. 5C). These results suggest that NPY-KO11 might exhibit higher stress response compared with NPY-KO7. The number of interaction with a mirror and time spent by the mirror was significantly lower in NPY-KO zebrafish than in wild-type fish (*p* < 0.05 in NPY-KO7 and KO11, F = 6.03, *p* < 0.05, one-way ANOVA; Fig. 5F, and F = 8.50, *p* < 0.01 one-way ANOVA; Fig. 5G, respectively). However, the total number and time for interaction with a mirror/swimming distance did not show the clear decrease of social interaction in NPY-KO unlike non-stress condition (Fig. 5H,I). Therefore, the NPY-KO zebrafish exhibited severe anxiety responses to acute stress, but the social interaction in NPY-KO showed small difference with wild type unlike non-stress condition.

### Black-white preference test

**Figure 6:**
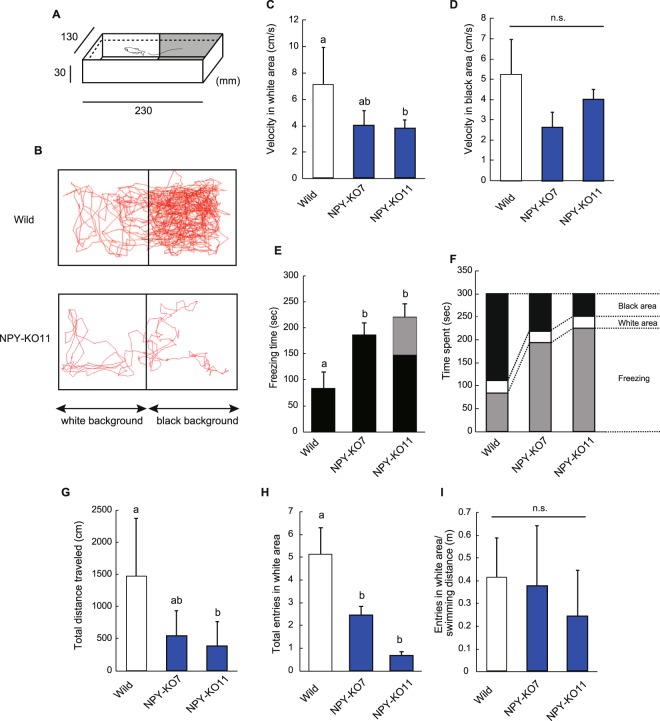
Changes in swimming behavior in black-white preference test. (**A**) Apparatus used in the black-white preference test. (**B**) Tracking of zebrafish swimming behaviour. (**C**,**D**) swimming velocities in the white area (**C**) and the black area (**D**). (**E**) Total time spent stationary (frozen). Black and grey colour indicate time of freezing in the black and white area, respectively. (**F**) Details of swimming behaviour in the tested period. Grey, white, and black colour indicate freezing, swimming in the white area, and the black area, respectively. (**G**) Total distance travelled. (**H**) Total number of entries into the white area. (**I**) Number of entries into the white area/swimming distance (m). N = 15. *n.s*., not significant. Columns having the same letter were not statistically different, *vice versa*.

The black-white reference test has been used to measure anxiety-like behaviours in zebrafish^33,34^. To test for anxiety-like behaviour further, the black-white preference test was conducted (Fig. 6A). In general, zebrafish prefer black to white colouration as it is more discreet, and white colour induces stress in zebrafish. In this test, entering a white area is used as an indicator of explorative behaviour. Although wild and NPY-KO zebrafish showed no abnormal behaviour during the acclimation, NPY-KO exhibited anxiety with the uncovering of white area after the removal of the wall between black and white area. As shown in Fig. 6B, NPY-KO zebrafish exhibited anxiety with the white area. The velocity of swimming speed of NPY-KO in white area was lower than wild type zebrafish (*p* < 0.05 in NPY-KO11) (F = 4.723, *p* < 0.05, one-way ANOVA; Fig. 6C), but not in the black area (Fig. 6D). NPY-KO froze significantly more than wild-type fish (NPY-KO7, *p* < 0.05; NPY-KO11, *p* < 0.01), (F = 7.15, *p* < 0.01, one-way ANOVA; Fig. 6E). Interestingly, KO7 and KO11 exhibited freezing behaviour in black and white area, which suggests zebrafish can recognize the colour away from the white zone. NPY-KO11 exhibited 33% of freezing in the white area while wild and NPY-KO7 did so only in black area (Fig. 6E), and swimming time spent in black area was decreased in NPY-KO7 and KO11 by the induction of freezing (Fig. 6F). Total distance travelled in NPY-KO was lower than wild zebrafish (p < 0.01 in NPY-KO11) (F = 7.00, p < 0.01, one-way ANOVA; (Fig. 6G). NPY-KO7 and -KO11 fish had significantly fewer entries into the white area (NPY-KO7, *p* < 0.05; NPY-KO11, *p* < 0.01) (F = 6.12, *p* < 0.01, one-way ANOVA; Fig. 6H), possibly due to the decrease of locomotion (Fig. 6I). These results suggest that exposure to the white area led NPY-KO to change their swimming behaviour.

### mRNA expression levels in the whole brain of NPY-KO zebrafish

**Figure 7:**
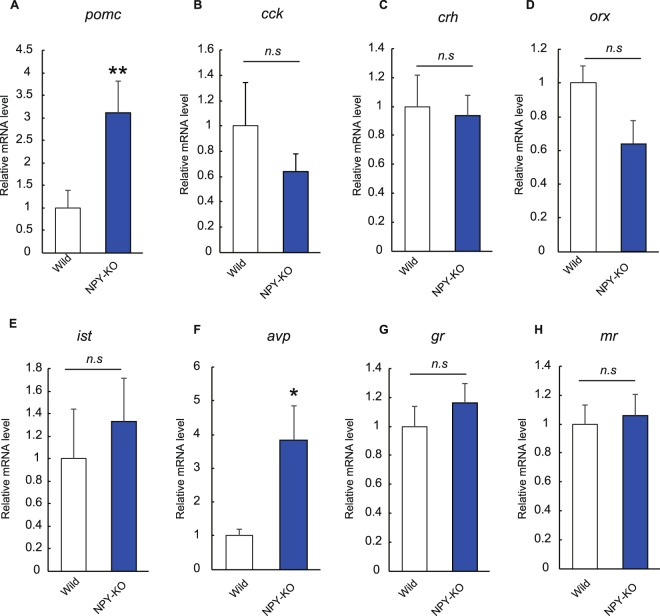
Changes in gene expression in NPY-KO zebrafish. mRNA levels of *pomc* (**A**), *cck* (**B**), *crh* (**C**), *orx* (**D**), *ist* (**E**), *avp* (**F**), *gr* (**G**) and *mr* (**H**) in wild-type and NPY-KO zebrafish. The mRNA level in NPY-KO zebrafish is shown as a relative value to that in wild-type fish in each gene analysis. N = 15. Results are shown as means ± standard errors of three independent experiments. *p < 0.05, **p < 0.01. *n.s*., not significant.

As several neuronal peptides regulate food intake and anxiety behaviour in zebrafish as they do in mammals^21,23^ and NPY induced the stress-resilience in rats^35^, stress- and anxiety-related gene expression levels were investigated in whole NPY-KO brains by real-time PCR of orexin (Orx), cholecystokinin (Cck), corticotropin-releasing hormone (Crh), arginine vasopressin (Avp), isotocin, (Ist, fish ortholog of oxytocin (Oxt)), pro-opiomelanocortin (Pomc), a glucocorticoid receptor (GR) and a mineralocorticoid receptor (MR). Orx, Cck, Crh, Ist and Avp are anxiety regulating peptide hormones, and Pomc is a precursor of adrenocorticotropic hormone and melanocyte-stimulating hormones (MSHs)^36^. Moreover, GR and MR mRNA levels are used as indicators of stress induction^37^. To investigate changes to orexigenic and anorexigenic hormones in NPY-KO zebrafish, GR and MR mRNA levels were estimated by real-time PCR. Among the anorexigenic peptides (*orx*, *cck*, *crh* and *pomc*), only the *pomc* mRNA level was significantly upregulated in NPY-KO zebrafish (*p* < 0.01) (Fig. 7A–D), despite there being no change in growth. We measured the mRNA levels of stress-related genes, such as *ist*, *avp*, *gr* and *mr*, in NPY-KO brains, and found that *avp* expression was significantly elevated in NPY-KO zebrafish (p < 0.05, Fig. 7E–H), indicating that NPY-KO zebrafish are chronically stressed even in normal condition.

**Figure 8:**
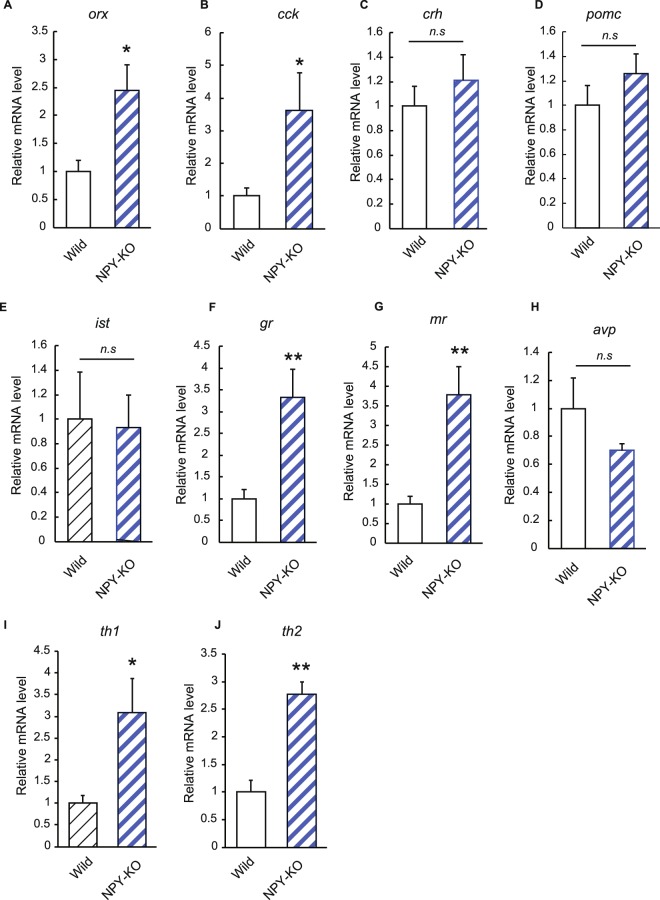
Changes in gene expression in NPY-KO zebrafish under acute stress. (**A**) *orx* mRNA level. (**B**) *cck* mRNA level. (**C**) *crh* mRNA level. (**D**) *pomc* mRNA level. (**E**) *ist* mRNA level. (**F**) *gr* mRNA level. (**G**) *mr* mRNA level. (**H**) *avp* mRNA level. (**I**) *th1* mRNA level. (**J**) *th2* mRNA level. Each gene expression level in NPY-KO zebrafish is relative to the value in wild-type fish. N = 15. Results are shown as means ± standard errors of three independent experiments. *p < 0.05, **p < 0.01. *n.s*., not significant.

To investigate the molecular mechanism underlying the increased anxiety observed in NPY-KO zebrafish under cold stress, the expression levels of anxiety and stress-related genes were estimated by real-time PCR. NPY-KO zebrafish had higher expression levels of two anxiety genes, *orx* and *cck*, than wild-type fish under cold stress (*p* < 0.05, Fig. 8A,B). Furthermore, the mRNA levels of two corticoid receptor genes, *gr* and *mr*, were higher than those in wild-type fish (4.1- and 4.9-fold, respectively, *p* < 0.01), although *crh*, *pomc* and *ist* expression levels were similar (Fig. 8C–G). Unlike under non-stress conditions (Fig. 8F), the *avp* mRNA level in NPY-KO zebrafish was almost the same as that in wild-type fish (Fig. 8H). In addition, the mRNA levels of tyrosine dehydroxylase 1 (*th1*) and 2 (*th2*), which are responsible for catecholamine induction, were significantly higher in NPY-KO zebrafish than in wild-type fish (*p* < 0.05 and *p* < 0.01, respectively) (Fig. 8I,J). Catecholamines, such as noradrenalin, induce fear and anxiety in mice^38^. These results indicate that knocking out NPY in zebrafish results in an increase in stress sensitivity accompanied by up-regulations of stress signals.

## Discussion

Recent studies have highlighted the multiple functions that NPY has in the central and peripheral nervous systems, its involvement in emotional behaviour, and NPY-related signalling as a target of psychiatry therapy. Zebrafish NPY is upregulated during handling stress^30^, and exogenous NPY injection into the brain increases food intake and suppresses anxious behaviour^21^. However, injecting exogenous NPY or a NPY receptor antagonist into the brain may not have long-lasting effects and may not reflect physiologically realistic levels, and may cause an overdose. To understand zebrafish NPY function under physiologically relevant conditions, we established two strains (NPY-KO7 and NPY-KO11) that had deletions of 7 and 11 nucleotides in *npy*, respectively. As there was no induction of mature NPY polypeptides in these mutants (Fig. 1H) and the mutants exhibited similar phenotypes throughout the study, NPY deficiency caused anxiety under stressful conditions.

Several reports have suggested that changes in fish behaviour in the mirror test represent negative social interactions, including aggression and anxiety, which are related to mammalian NPY functions^39,40^. While the NPY-KO zebrafish did not exhibit any freezing in the mirror test, they interacted with the mirror less often and in a shorter time than wild-type fish and upregulated their *avp* and *pomc* expression levels, suggesting that NPY deficiency decreases social interactions, including aggression. In zebrafish, *avp* mRNA is involved in hierarchical relationships, and an intraperitoneal injection of Avp suppresses social behaviour^41^. Furthermore, in mammals, NPY expression in brain regions such as the olfactory bulb, the hypothalamus and the amygdala is involved in the regulation of aggression^40^. The mouse Y1 receptor increases aggressive behaviour, suggesting that the effects of NPY on aggression are related to serotonergic signalling via the 5-HT1A receptor^42^. Zebrafish also possess a Y1 receptor gene in their genomes, and a co-ICV injection of NPY and mammalian Y1 antagonist BIBP-3226 suppresses fish Y1 receptor functioning, such as suppression of anxiety in black-white test^21,23^. Therefore, zebrafish NPY mutants may be less aggressive than wild-type zebrafish because of Y1 and Avp signalling.

Acute stress caused the NPY-KO zebrafish to exhibit anxiety behaviours such as freezing, decreasing movement and velocity, and swimming along the side of the tank. These are typical anxiety behaviours in zebrafish. Anxiety was also observed in the black-white preference test. Similar phenotypes have been observed in NPY-knockout mice in field tests^6^. In mammals, acute stress increases *npy* mRNA expression via a negative feedback pathway through corticoid/corticoid receptor pathways. We found that acute cold stress caused *npy* upregulation and the NPY-KO zebrafish exhibited large increases in *mr* and *gr* expression, indicating that they were stressed. Interestingly, *crh* and *pomc* expression levels in NPY-KO zebrafish were unaffected under stressful conditions. In mammals, Oxt and Avp/Crh, as well as NPY/Crh, are involved in stress reduction^43^, and we found that *ist* and *avp* mRNA levels were similar in wild-type and NPY-KO zebrafish. This suggests that this pathway did not compensate for the lack of NPY under stressful conditions. However, the anxiogenic genes *orx* and *cck* were upregulated in the NPY-KO zebrafish under stress. Orexin is an anxiogenic neuropeptide in mammals, and orexin-containing cells are innervated by NPY and αMSH-containing arcuate neurons^44^. Mouse orexin and noradrenergic neurons mediate fear-related behaviours such as freezing, which is sometimes seen in psychiatric disorders such as post-traumatic stress disorder^38^. In goldfish, an ICV administration of orexin causes an anxiogenic response that is decreased by SB334867, which is an antagonist of orexin receptor 1 (OX1R)^45^. NPY deficiency may upregulate orexin and activate noradrenergic neurons in NPY-KO zebrafish, which causes freezing. We found that NPY-KO zebrafish under acute stress exhibited freezing accompanied with an upregulation of *th1* and *th2*, suggesting the involvement of catecholamines in NPY-KO anxiety behaviour. Recently, it has been reported that *npy* mutant zebrafish are more active and sleep less during the day, which is controlled by noradrenergic signalling^22^. In SK-N-MC neuroblastoma cells, NPY decreases tyrosine hydroxylase mRNA levels^46^. Further work is required to investigate the mechanism underlying freezing behaviour in NPY-KO zebrafish.

In summary, NPY-KO zebrafish exhibited less social behaviour under non-stressful conditions than wild-type fish, whereas under acute stress, they exhibited severe anxiety, similar to NPY-knockout mice and human psychiatric patients. Unlike previous studies of anxiety in zebrafish that used chemicals such as alcohol^47^, caffeine^48^ and Y1 blockers^21^, anxiety behaviours were easily inducible and long-term observations were possible in NPY-KO zebrafish. These unique characteristics of NPY-KO zebrafish make them suitable experimental animals for studying psychiatric disorders.

## Methods

### Animals

Zebrafish *RIKEN WT* (RW) strain was obtained from the Centre for Brain Science, Institute of Physical and Chemical Research (RIKEN), Japan. Zebrafish were housed in a 3-L water tank (MEITO system, Japan) with a recirculating filtration system and UV sterilization under a 14/10 h light/dark cycle at 28 °C. Live brine shrimp and a commercial diet (Otohime B2, Marubeni Nisshin Feed Ltd., Tokyo, Japan) were provided twice a day. For breeding, a pair (male and female) was transferred to a 1.3-L breeding tank.

### Ethics approval

All protocols for this study were approved by the Kagoshima University Committee for Animal Experiments and performed in accordance with relevant guidelines and regulations.

### Establishment of *npy*-knockout zebrafish by CRISPR/Cas9

Establishing the zebrafish *npy*-deficient mutant line (NPY-KO) was performed by microinjecting recombinant Cas9 protein (rCas9, New England BioLabs, MA, USA), tracrRNA and *npy*-specific single gRNAs. To avoid off-target effects, a 21-nt RNA sequence adjacent to a protospacer-adjacent motif (PAM) in *npy* was selected for designing specific gRNAs using CRISPRdirect (https://crispr.dbcls.jp/). gRNA#1 (5′-UUCUCUUGUUCGUCUGCUUGguuuuagagcuaugcuguuuug-3′), gRNA#2 (5′-GCGUCCUCUCCCGGGUUGUCguuuuagagcuaugcuguuuug-3′) and tracrRNA (5′-AAACAGCAUAGCAAGUUAAAAUAAGGCUAGUCCGUUAUCAACUUGAAAAAGUGGCACCGAGUCGGUGCU-3′) were synthesized by FASMAC (Japan). One nanolitre of rCas9/tracrRNA/crRNA complex (100 nM rCas9, 200 pg/µL tracrRNA and 100 ng/µL crRNA) was microinjected into zebrafish 1-cell-stage embryos using a micromanipulator MN-153 (Narishige, Japan).

A HMA was run to detect genomic DNA mutations in *npy*. Genomic DNA was extracted from zebrafish whole embryos or tail fins and partial *npy* DNA fragments, including a crRNA-targeting sequence, were amplified by PCR with KOD plus NEO (TOYOBO, Japan) and the primers (5′-AAGATGTGGATGAGCTGGGC-3′) and (5′-TGAATAATACTTGGCGAGCTCCT-3′) using the following protocol: 35 cycles at 95 °C for 10 s and 68 °C for 20 s. The resulting PCR products were subjected to annealing and electrophoresis on 12% polyacrylamide gels. To distinguish NPY-KO zebrafish from wild-type zebrafish, the PCR products were mixed with PCR products from wild-type fish and annealed for HMA. PCR products were then sub-cloned into pBluescript plasmid and some of their nucleotide sequences were analysed using a ABI3130xl Genetic Analyzer (Life Technologies, MA, USA). F0 was crossed with WT fish to generate F1. F1 possessing same *npy* mutation (5 or 11-deletion in *npy* gene) were intercrossed for the establishment of F2 generation. F2 generations was co-housed until genotyping and the genotyping was carried out before behavioural analysis.

To confirm the transcription of mutated *npy* RNA in the NPY-KO zebrafish, primers specific to intact *npy*, but not to mutated *npy*, were designed for PCR. Total RNA was extracted from wild-type and NPY-KO brains using Sepasol-RNA I Super G (Nacalai Tesque, Japan) followed by cDNA synthesis using ReverTra Ace qPCR RT Master Mix with gDNA Remover (TOYOBO). Partial *npy* cDNA was amplified by PCR with KOD plus NEO (TOYOBO) and the primers (5′-TTCTCTTGTTCGTCTGCTTGG-3′) and (5′-ATATCTGGTCTGGGGGCGGG-3′) using the following protocol: 35 cycles at 95 °C for 10 s and 68 °C for 20 s. A PCR for *actb* was also conducted using the same cDNAs to confirm the RNA extraction and reverse transcription. Obtained mutants (F2 generations) and their siblings were used as NPY-KO and “Wild” in this study, respectively.

### Immunohistochemistry

The 48-h fasted zebrafish were perfused with 4% paraformaldehyde in 0.1 M phosphate buffer. Brain sections (14 µm) of the diencephalon were treated with 3% H_2_O_2_ in PBS for 15 min, and 1% BSA and 0.2% Triton X-100 in PBS for 30 min. And then, the sections were incubated with a primary antibody against NPY (N9528, Sigma-Aldrich, St Louis, MO, 1:5000) overnight at room temperature, with biotinylated anti-goat IgG solution (BA1000, Vector Labs, Burlington, CA, 1:200) for 3 h at room temperature, and with Vectastain ABC reagents (Vector Laboratories, Burlingame, CA) for 1 h at room temperature. The sections were visualized with 0.02% diaminobenzidine tetrahydrochloride and 0.005% H_2_O_2_ in 0.05 M Tris-HCl buffer for 20 min and observed using a light microscope (BX51, Olympus Optical Co. Ltd., Tokyo, Japan). Nissl bodies were counterstained with 0.5% cresyl violet solution.

### Mirror test

A mirror test was conducted as described elsewhere, with slight modifications^39^. The tank was 5 cm high, 10 cm wide and 24 cm long, and a mirror was placed on one side (Fig. 4A). A fish (6-month-old) was placed on the opposite side of the mirror side and its behaviour recorded for 10 min using a digital video camera, which was analysed using Move-tr/2D software (Library, Japan). When fish approached to the mirror, its angle is perpendicular. After approaching, fish exhibited two responses: (i) Fish parallelly swims along with the opponent in the mirror (defined as interaction), or (ii) Fish turns back without interaction. In this study, (i) was defined as social behaviour and estimated the difference between wild and KO. The total number of interaction and the amount of time of the interaction, and the total distance travelled were recorded.

Next, to investigate the effect of acute stress on zebrafish social behaviour, fish were exposed to cold water which induces acute stress in zebrafish^49^. Fish (6-month-old) were placed in water that was 10 °C for 2 s, before being immediately transferred to the tank used in the mirror test, as described above. The fish were tracked for 5 min, and the number of interaction with the mirror and its time spent at the mirror, freezing occurrences, total distance travelled and time spent within 3 cm of the sides of the tank were recorded using Move-tr/2D software.

### Black-white preference test

The trial was designed based on Mazimino *et al*.^50^, with some modifications. A fish tank was divided into two compartments, black and white, that were 3 cm high, 13 cm wide and 23 cm long, and were separated by a removable wall. Firstly, fish (6-month-old) were placed in the black compartment for 10 min acclimation. Subsequently, after removing the dividing wall, locomotion was tracked by a video camera for 5 min. The number of entries into the white compartment, velocity, total distance travelled, time spent stationary (frozen) and fish tracking were recorded using Move-tr/2D software. Freezing was defined as the complete cessation of movements with the exception of the eye and operculate movements^51^.

### Real-time PCR

The mRNA expression levels of various genes were analysed using zebrafish whole brain cDNAs using a StepOne Real-Time PCR System (Thermo Fisher Scientific, MA, USA). The PCR was conducted using KOD SYBR Green PCR Master Mix (TOYOBO) and the primers shown in Supplemental Table 1. DNA amplification was quantified from the C (T) value based on standard curves to ensure quantification was within a linear range. As it is reported that *actb* expression is not altered by acute stress^52^, the expression level of *actb* mRNA was used for gene expression normalization. For the estimation of the alteration of gene expression by cold stress, fish were placed in water that was 10 °C for 2 s and then moved to a normal tank. At 15 min after exposure, fish were anesthetized and used for RNA extraction.

### Data analysis

Results are expressed as means ± standard errors, and all values were compared by Student’s *t*-test. Comparisons between two groups were performed using two-tailed Student’s *t*-tests. One-way or two-way analysis of variance (ANOVA) followed by Tukey’s multiple comparison test was used to compare three or more groups.

## Supplementary information

Supplementary information.

## References

[1] Gehlert DR (2004). Introduction to the reviews on neuropeptide Y. Neuropeptides.

[2] Inui A (1999). Feeding and body-weight regulation by hypothalamic neuropeptides - Mediation of the actions of leptin. Trends in Neurosciences.

[3] Sah R, Geracioti TD (2013). Neuropeptide Y and posttraumatic stress disorder. Molecular Psychiatry.

[4] Reichmann F, Holzer P (2016). Neuropeptide Y: A stressful review. Neuropeptides.

[5] Gøtzsche CR, Woldbye DPD (2016). The role of NPY in learning and memory. Neuropeptides.

[6] Bannon AW (2000). Behavioral characterization of neuropeptide Y knockout mice. Brain Research.

[7] Mickey BJ (2011). Emotion processing, major depression, and functional genetic variation of neuropeptide Y. Archives of General Psychiatry.

[8] Redrobe JP, Dumont Y, Quirion R (2002). Neuropeptide Y (NPY) and depression: From animal studies to the human condition. Life Sciences.

[9] Domschke K (2010). Neuropeptide Y (NPY) gene: Impact on emotional processing and treatment response in anxious depression. European Neuropsychopharmacology.

[10] Karl T, Burne THJ, Herzog H (2006). Effect of Y1 receptor deficiency on motor activity, exploration, and anxiety. Behavioural Brain Research.

[11] Mione MC, Trede NS (2010). The zebrafish as a model for cancer. Disease Models & Mechanisms.

[12] Oka T (2010). Diet-induced obesity in zebrafish shares common pathophysiological pathways with mammalian obesity. BMC Physiology.

[13] Ramesh T (2010). A genetic model of amyotrophic lateral sclerosis in zebrafish displays phenotypic hallmarks of motoneuron disease. Disease Models & Mechanisms.

[14] Dreosti E, Lopes G, Kampff AR, Wilson SW (2015). Development of social behavior in young zebrafish. Frontiers in Neural Circuits.

[15] Howe K (2013). The zebrafish reference genome sequence and its relationship to the human genome. Nature.

[16] Kalueff AV, Stewart AM, Gerlai R (2014). Zebrafish as an emerging model for studying complex brain disorders. Trends in Pharmacological Sciences.

[17] Zon LI, Peterson RT (2005). *In vivo* drug discovery in the zebrafish. Nature Reviews Drug Discovery.

[18] Parng C, Seng WL, Semino C, McGrath P (2002). Zebrafish: a preclinical model for drug screening. Assay and drug development technologies.

[19] Green J (2012). Automated high-throughput neurophenotyping of zebrafish social behavior. Journal of Neuroscience Methods.

[20] Söderberg C (2000). Zebrafish genes for neuropeptide Y and peptide YY reveal origin by chromosome duplication from an ancestral gene linked to the homeobox cluster. Journal of Neurochemistry.

[21] Yokobori E (2012). Neuropeptide Y stimulates food intake in the zebrafish, Danio rerio. Journal of Neuroendocrinology.

[22] Singh C, Rihel J, Prober DA (2017). Neuropeptide Y regulates sleep by modulating noradrenergic signaling. Current Biology.

[23] Matsuda K, Sakashita A, Yokobori E, Azuma M (2012). Neuroendocrine control of feeding behavior and psychomotor activity by neuropeptideY in fish. Neuropeptides.

[24] Sundström G, Larsson TA, Xu B, Heldin J, Larhammar D (2013). Interactions of zebrafish peptide YYb with the neuropeptide Y-family receptors Y4, Y7, Y8a, and Y8b. Frontiers in Neuroscience.

[25] Naito Y, Hino K, Bono H, Ui-Tei K (2015). CRISPRdirect: Software for designing CRISPR/Cas guide RNA with reduced off-target sites. Bioinformatics.

[26] Gallup GG (1970). Chimpanzees: Self-recognition. Science.

[27] Reiss, D. & Marino, L. Mirror self-recognition in the bottlenose dolphin: A case of cognitive convergence. *Proceedings of the National Academy of Sciences of the United States of America* **98**, 5937–5942 (2001). 10.1073/pnas.101086398 PMC33317 11331768

[28] Way GP, Ruhl N, Snekser JL, Kiesel AL, McRobert SP (2015). A comparison of methodologies to test aggression in zebrafish. Zebrafish.

[29] Flemming A (2007). Stress and obesity connect at NPY. Nature Reviews Drug Discovery.

[30] Cortés R (2018). Effects of acute handling stress on short-term central expression of orexigenic/anorexigenic genes in zebrafish. Fish Physiology and Biochemistry.

[31] Conde-Sieira, M., Chivite, M., Míguez, J. M. & Soengas, J. L. Stress effects on the mechanisms regulating appetite in teleost fish. *Frontiers in Endocrinology* **9** (2018). 10.3389/fendo.2018.00631 PMC6205965 30405535

[32] Donaldson MR, Cooke SJ, Patterson DA, Macdonald JS (2008). Cold shock and fish. Journal of Fish Biology.

[33] Córdova SD, dos Santos TG, de Oliveira DL (2016). Water column depth and light intensity modulate the zebrafish preference response in the black/white test. Neuroscience Letters.

[34] Blaser, R. E. & Rosemberg, D. B. Measures of anxiety in zebrafish (*Danio rerio*): Dissociation of black/white preference and novel tank test. *Plos one* **7**, e36931 (2012). 10.1371/journal.pone.0036931 PMC3355173 22615849

[35] Silveira Villarroel H (2018). NPY induces stress resilience via downregulation of Ih in principal neurons of rat basolateral amygdala. The Journal of Neuroscience.

[36] Ataka K, Nagaishi K, Asakawa A, Inui A, Fujimiya M (2012). Alteration of antral and proximal colonic motility induced by chronic psychological stress involves central urocortin 3 and vasopressin in rats. American Journal of Physiology-Gastrointestinal and Liver Physiology.

[37] Veenema AH, Meijer OC, De Kloet ER, Koolhaas JM, Bohus BG (2003). Differences in basal and stress-induced HPA regulation of wild house mice selected for high and low aggression. Hormones and Behavior.

[38] Soya S (2017). Orexin modulates behavioral fear expression through the locus coeruleus. Nature Communications.

[39] Nunes ME (2017). Chronic treatment with paraquat induces brain injury, changes in antioxidant defenses system, and modulates behavioral functions in zebrafish. Molecular Neurobiology.

[40] Karl T, Herzog H (2007). Behavioral profiling of NPY in aggression and neuropsychiatric diseases. Peptides.

[41] Filby, A. L., Paull, G. C., Hickmore, T. F. A. & Tyler, C. R. Unravelling the neurophysiological basis of aggression in a fish model. *BMC Genomics* **11**, 498 (2010). 10.1186/1471-2164-11-498 PMC2996994 20846403

[42] Karl T (2004). Y1 receptors regulate aggressive behavior by modulating serotonin pathways. Proceedings of the National Academy of Sciences of the United States of America.

[43] Kormos V, Gaszner B (2013). Role of neuropeptides in anxiety, stress, and depression: From animals to humans. Neuropeptides.

[44] Alpar A, Harkany T (2013). Orexin neurons use endocannabinoids to break obesity-induced inhibition. Proceedings of the National Academy of Sciences of the United States of America.

[45] Nakamachi T (2014). Orexin A enhances locomotor activity and induces anxiogenic-like action in the goldfish, Carassius auratus. Hormones and Behavior.

[46] Cavadas C (2006). Deletion of the neuropeptide Y (NPY) Y1 receptor gene reveals a regulatory role of NPY on catecholamine synthesis and secretion. Proceedings of the National Academy of Sciences of the United States of America.

[47] Baiamonte M, Parker MO, Vinson GP, Brennan CH (2016). Sustained effects of developmental exposure to ethanol on zebrafish anxiety-like behaviour. Plos one.

[48] Egan RJ (2009). Understanding behavioral and physiological phenotypes of stress and anxiety in zebrafish. Behavioural brain research.

[49] Wu CL (2011). Zebrafish HSC70 promoter to express carp muscle-specific creatine kinase for acclimation under cold condition. Transgenic Research.

[50] Maximino C (2010). Parametric analyses of anxiety in zebrafish scototaxis. Behavioural Brain Research.

[51] Maximino C, Puty B, Matos Oliveira KR, Herculano AM (2013). Behavioral and neurochemical changes in the zebrafish leopard strain. Genes, Brain and Behavior.

[52] Lefebvre KA (2009). Gene expression profiles in zebrafish brain after acute exposure to domoic acid at symptomatic and asymptomatic doses. Toxicological Sciences.

