# Supplementary material for: Neuropeptide Y deficiency induces anxiety-like behaviours in zebrafish (Danio rerio)
Source: Sci Rep. 2020 Apr 3;10:5913. doi: 10.1038/s41598-020-62699-0 (PMC7125123; doi:10.1038/s41598-020-62699-0)
Supplement: Supplementary file 1 — Supplementary information. [file 41598_2020_62699_MOESM1_ESM.pdf]

Neuropeptide Y deficiency induces anxiety-like behaviours in zebrafish (*Danio rerio*)

Kazuhiro Shiozaki [a, b, *], Momoko Kawabe [a], Kiwako Karasuyama [a], Takayoshi Kurachi [a], Akito Hayashi [a], Koji Ataka [c], Haruki Iwai [d], Hinako Takeno [a], Oki Hayasaka [b], Tomonari Kotani [a, b], Masaharu Komatsu [a, b], Akio Inui [c]

[a] Department of Food Life Science, Faculty of Fisheries, Kagoshima University, Kagoshima, Japan

[b] The United Graduate School of Agricultural Sciences, Kagoshima University, Kagoshima, Japan

[c] Department of Pharmacological Sciences of Herbal Medicine, Graduate School of Medical and Dental Sciences, Kagoshima University, Kagoshima, Japan

[d] Department of Oral Anatomy and Cell Biology, Graduate School of Medical and Dental Sciences, Kagoshima University, Kagoshima, Japan

***Corresponding author:** Kazuhiro Shiozaki

4-50-20 Shimoarata, Kagoshima 890-0056, Japan. tel/fax: +81 99 286 4170, E-mail:shiozaki@fish.kagoshima-u.ac.jp (K.Shiozaki)

Supplemental Table 1 Primers used in the study.

Supplemental Fig. 1. Nucleotide and deduced amino acid sequence of the zebrafish NPY. Nucleotide sequence of zebrafish *npy* was obtained from Genbank (accession No: NM131074). Exon/intron boundary is represented by vertical lines. Red coloured and underlined characters indicate mature NPY amino acid sequence and gRNA-targeted region, respectively.

Supplemental Fig. 2. Full length gels of electrophoresis for the detection of NPY mutant in zebrafish. (A-C) Full length gels in HMA analysis of F0 shown in Fig. 1B (A), F1 in Fig. 1D (B) and F2 in Fig. 1F (C). (D) Full length of gel to detect expression of intact *npy* mRNA in wild-type fish and mutants shown in Fig. 1G (F2). cDNAs from wild-type fish and mutants were used for PCR as a template, using primers specific to intact *npy. actb* was used as an internal control.

Supplemental Fig. 3. Sequencing of *npy* mRNA in wild and NPY-KO. The nucleotide sequence of the gRNA-target region in *npy* was sequenced in wild (upper panel), NPY-KO7 (middle panel) and NPY-KO11 (lower panel).

Supplemental Fig. 4. Distribution of NPY-neurons in wild and NPY-KO zebrafish brain.

NPY signals were immuno-stained by NPY-specific antibody and DAB staining. Neuronal cells were detected by Nissl staining. The area around the ventral zone of the periventricular hypothalamus (HV), anterior tuberal nucleus (ATN) and lateral hypothalamic areas (LH) were highlighted with red circle. Black bar indicates 100 μm.

Supplemental table 1 Real-time PCR primers used in this study

Gene	Primers	
orx	5´- TTCATGGCGCTGCTAGCTCA -3´	5´- AATTTAGCGGGCTCCTCCAGC -3´
cck	5´- AGCGTCAACCACCGGATAAA -3´	5´- AGAGGACAGACGGAAACACG -3´
crh	5´- TCGTCCATGATCTTGCGGTT -3´	5´- GAATCTGCACGTGGTTGTCG -3´
pomc	5´- CCCCCTACAAAATGACCCAT -3´	5´- ATCCTTCCTCGGTTGGTCTT -3´
avp	5´- CGCTCTCGTCTGCCTGCTAC -3´	5´- TCTTAAGTCCCGCGCTGCTG -3´
gr	5´- CCCATTGAGGACCAAATCAC -3´	5´- AGTAGAGCATTTGGGCGTTG -3´
mr	5´- TTCGGACTAAAAACGCTGGACG -3´	5´- TCGCCGCAGTCTGAGGTCGT -3´
ist	5´- TCTGGAAAGGCCTGCGGTTA -3´	5´- GCTGTTGGCCGGTTGATTGA -3´
th1	5´- TACATACGGCACGCTTCCTC -3´	5´- GAACCGCACAGAAAACGGTC -3´
th2	5´- AAAGGCTTATGGGGCTGGAC -3´	5´- GCTGCAAGTGTAGGGGTCAT -3´
npy	5´- AAGATGTGGATGAGCTGGGC -3´	5´- TGAATAATACTTGGCGAGCTCCT -3´
actb	5´- AGCACCCTGTGCTGCTCACT -3´	5´- CGCCATACAGAGCAGAAGCCA -3´

```
          10         20         30         40         50         60
    cccacagagcaagaattccaatcaagatctcaaaaaattccaagacctcattcactgatg
  1                                                            M     20
          70         80         90        100        110        120
    aatccaaacatgaagatgtggatgagctgggcagcgtgcgcgtttctcttgttcgtctgc
 21 N  P  N  M  K  M  W  M  S  W  A  A  C  A  F  L  L  F  V  C      40
         130        140        150        160        170        180
    ttggggactctcacagaagggtatccaacaaaacccgacaacccgggagaggacgcacct
 41 L  G  T  L  T  E  G  Y  P  T  K  P  D  N  P  G  E  D  A  P      60
         190        200        210        220        230        240
    gcggaggagctcgccaagtattattcagcactaagacactacatcaacctcataacaagg
 61 A  E  E  L  A  K  Y  Y  S  A  L  R  H  Y  I  N  L  I  T  R      80
         250        260        270        280        290        300
    cagaggtatgggaaaaggtcaagcgctgacaccttaatttcagaccttctgattggtgaa
 81 Q  R  Y  G  K  R  S  S  A  D  T  L  I  S  D  L  L  I  G  E     100
         310        320        330        340        350        360
    acagagtcccgcccccagaccagatatgaggatcatttggcatggtgatctcatccactc
101 T  E  S  R  P  Q  T  R  Y  E  D  H  L  A  W  *                  120
         370        380        390        400        410        420
    tgtgatgtccatgtgtccttctgttaacaattttggccaccagatctcataaaactcaaa
    tgttctgcataaacaacttgctgtgacacaccacagtcatcatcaggatagtgaaaatac
    tgcagaatgttcaactaacacattacccataatgtctgtacaaatacgtcaatgtgcgc
    tctttaggctcatcgaagattcagttcaacaagacagttgtacagagactcattttgtgt
    atgcgtgttgtgtccattgtaatcccaagtagcaaaggagaacaataatgactgtacagt
    acaacagtgctaataaagatcactatatttcaaagagtcgtctttgtttgctgtcttttg
    cccctga
```

Supplemental Fig. 1.

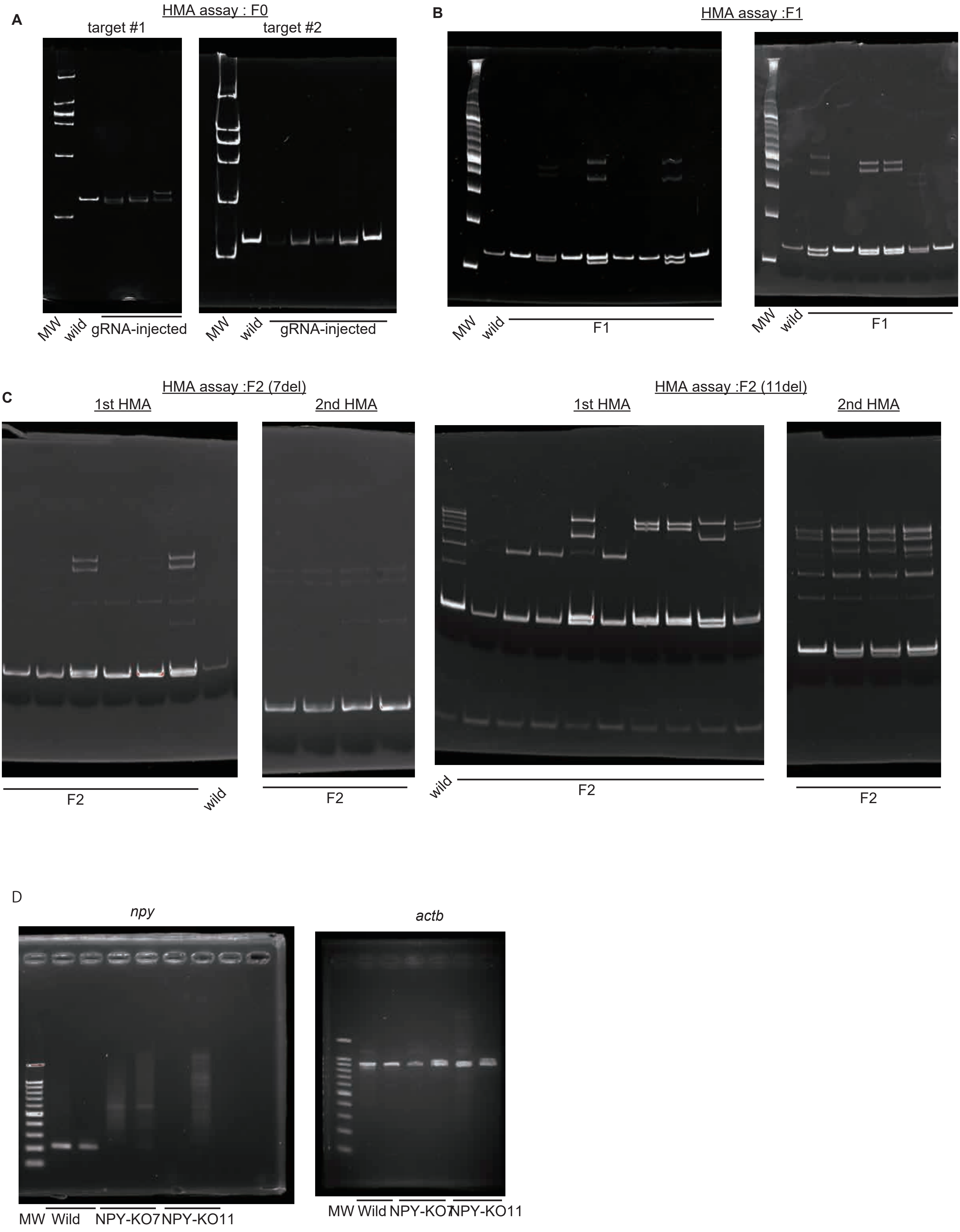

Supplemental Fig. 2.

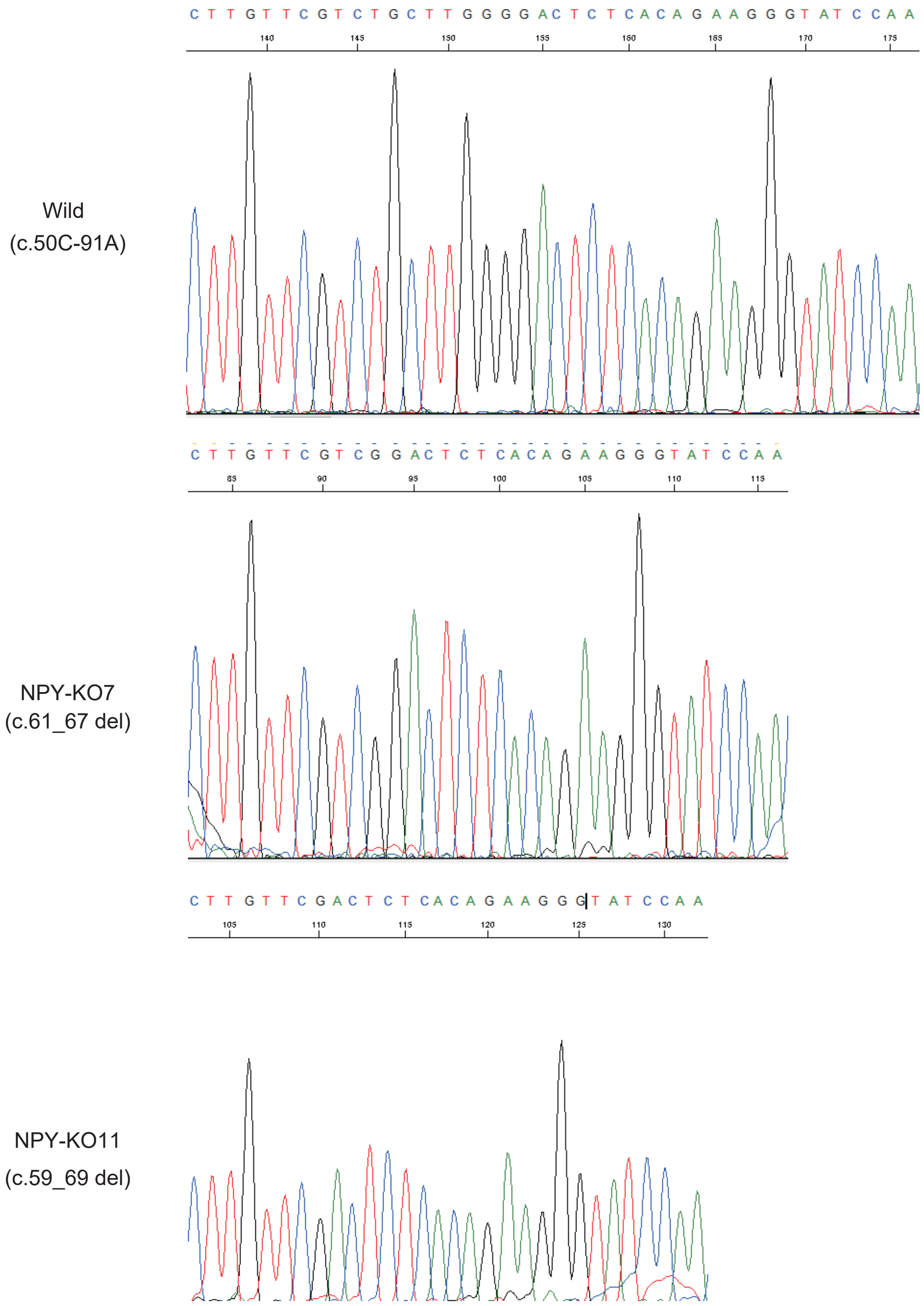

Supplemental Fig. 3.

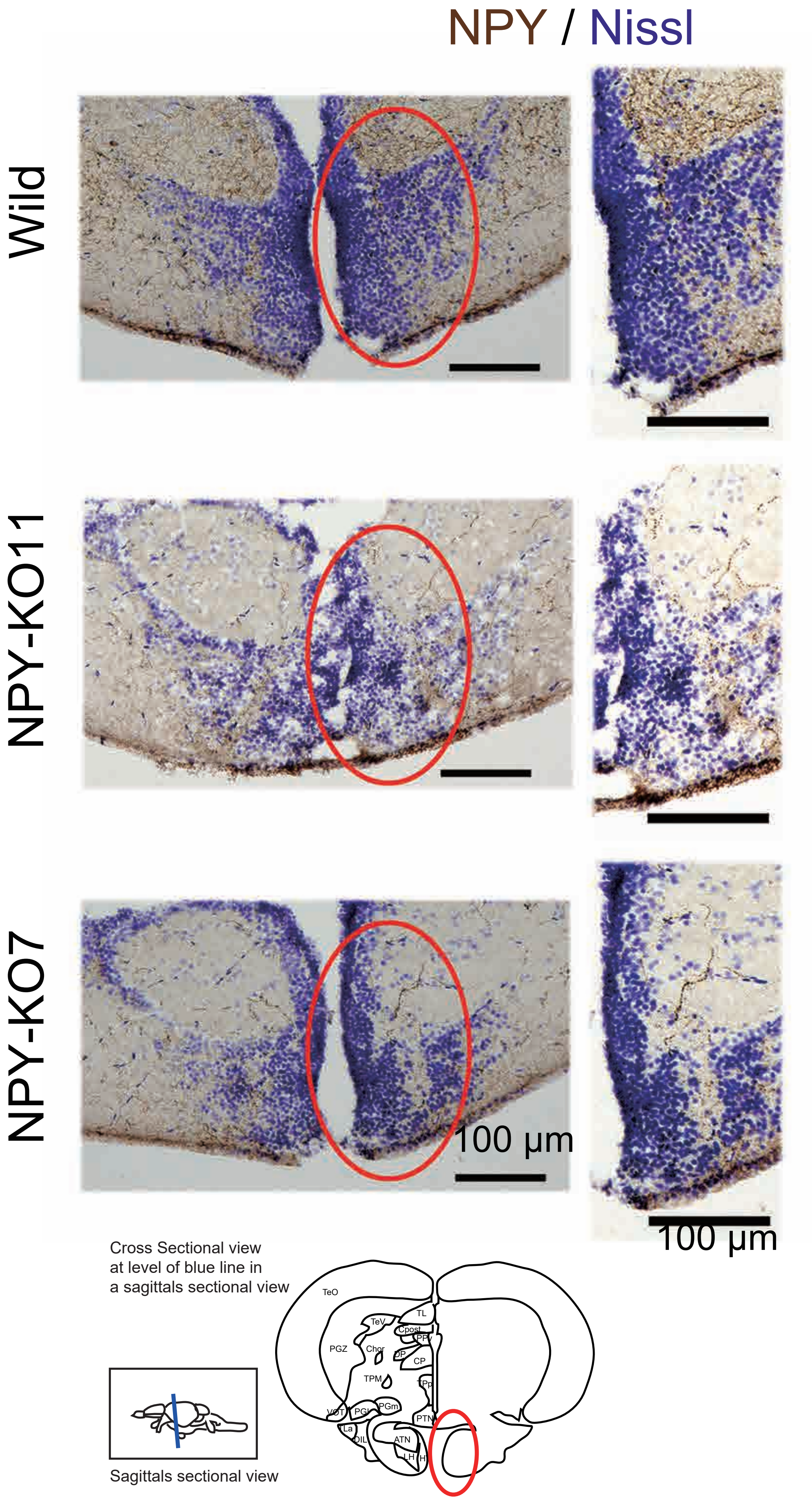

Supplemental Fig. 4
